# Diffusion-Based Separation of Extracellular Vesicles by Nanoporous Membrane Chip

**DOI:** 10.3390/bios11090347

**Published:** 2021-09-19

**Authors:** Gijung Kim, Min Chul Park, Seonae Jang, Daeyoung Han, Hojun Kim, Wonjune Kim, Honggu Chun, Sunghoon Kim

**Affiliations:** 1 Department of Biomedical Engineering, Korea University, Seoul 02841, Korea; novelty@korea.ac.kr (G.K.); hjkim1017@korea.ac.kr (H.K.); aether26@korea.ac.kr (W.K.); 2 Medicinal Bioconvergence Research Center, Suwon 16229, Korea; minchul.park@neomics.com (M.C.P.); ladin20@snu.ac.kr (D.H.); 3 Department of Biomicrosystem Technology, Korea University, Seoul 02841, Korea; suneai87@hanmail.net; 4 Interdisciplinary Program in Precision Public Health, Korea University, Seoul 02841, Korea; 5 Institute for Artificial Intelligence and Biomedical Research, Seoul 03722, Korea; 6 College of Pharmacy & College of Medicine, Gangnam Severance Hospital, Yonsei University, Seoul 03722, Korea

**Keywords:** exosomes, diffusion-based separation, PC membrane, nanopore

## Abstract

Extracellular vesicles (EVs) have emerged as novel biomarkers and therapeutic material. However, the small size (~200 nm) of EVs makes efficient separation challenging. Here, a physical/chemical stress-free separation of EVs based on diffusion through a nanoporous membrane chip is presented. A polycarbonate membrane with 200 nm pores, positioned between two chambers, functions as the size-selective filter. Using the chip, EVs from cell culture media and human serum were separated. The separated EVs were analyzed by nanoparticle tracking analysis (NTA), scanning electron microscopy, and immunoblotting. The experimental results proved the selective separation of EVs in cell culture media and human serum. Moreover, the diffusion-based separation showed a high yield of EVs in human serum compared to ultracentrifuge-based separation. The EV recovery rate analyzed from NTA data was 42% for cell culture media samples. We expect the developed method to be a potential tool for EV separation for diagnosis and therapy because it does not require complicated processes such as immune, chemical reaction, and external force and is scalable by increasing the nanoporous membrane size.

## 1. Introduction

Extracellular vesicles (EVs), which play key roles in intercellular communication between distant cells, are classified by their sizes and biogenesis mechanisms [1]. For example, exosomes, originated from multivesicular endosome fusion, are vesicles ranging from 50 nm to 150 nm in diameter [2]. Apoptotic bodies, originated from apoptosis, range from 300 nm to 5000 nm in diameter [3]. Exosomes contain genetic molecules, such as DNA, microRNA, and mRNA, and proteomic/metabolomic molecules, such as protein and lipid [4,5,6]. These biomolecules inside and on the surface of the exosomes perform several biological functions, e.g., intercellular communication, immune responses, and apoptosis [7,8,9]. Furthermore, exosomes are involved in the formation of cancer [10,11,12], organotrophic metastasis of cancer [13,14,15], and neurodegeneration [16,17,18]. It has been continuously reported that exosomes are closely related to several other diseases [19,20,21]; accordingly, many studies are underway to utilize exosomes as diagnostic biomarkers. Moreover, exosomes can be used for therapeutic purposes, such as drug delivery by loading drugs inside or on the surface of the exosome [22,23,24]. EV separation methods currently being used are ultracentrifugation [25], density gradient centrifugation [26], immuno-chemical separation [27], size exclusion chromatography [28], and microfluidics-based separations [29,30]. Despite the tremendous interest in exosome diagnosis and therapeutics, no standard method has been established yet to isolate highly pure exosomes while retaining their chemical and physical properties [20,31,32,33,34]. In addition, clinical and industrial applications require simple operation and scalability for high-throughput separation. Efficient separation methods that fulfill these requirements are highly desirable [35].

Among the above-mentioned EV isolation methods, ultracentrifugation is the most widely used for obtaining exosomes without chemical addition. However, it shows a low recovery rate and low physical intactness of exosomes due to a high g force (~100,000 g) that corresponds to the 101 bar of hydrostatic pressure [36] during sedimentation [37,38,39]. We contemplated that diffusion-based exosome separation using a nanopore membrane would be advantageous to obtain intact exosomes in high-throughput without chemical addition. To compare the separation speed of diffusion-based separation with ultracentrifugation, the mathematical model for each method was derived. The model for analyzing sedimentation is closely related to the particle size, density, and viscosity of the fluids. The equation for spherical particle sedimentation velocity in centrifuge-based separation is given as follows [40,41,42]:

(1) $$V=\frac{6g\left(\rho_{p}-\rho_{f}\right)r^{2}}{\eta }$$

where *g*, *ρ_p_*, *ρ_r_*, *r*, and *η* are the gravitational acceleration, density of the particle, density of the fluid, radius of a spherical particle, and dynamic viscosity of the fluid, respectively. The sedimentation velocity is a function of the *r*^2^, resulting in separation that is efficient for large (>1 μm) particles [43]. On the other hand, diffusion of particles in solution can be analyzed with the kinetic theory of gases, and the general form is [44]

(2) $$D=\mu k_{B}T$$

where *D*, *μ*, *k**_B_***, and *T* are the diffusion coefficient, the ratio of the particle’s drift velocity to an applied force, Boltzmann’s constant, and absolute temperature, respectively. The Equation (2) is modified to describe the diffusing spherical particles in a liquid, and it is the Stokes–Einstein equation that is widely used [45]

(3) $$D=\frac{k_{B}T}{6\pi \eta r}$$

where *η* and *r* are the dynamic viscosity and radius of the spherical particle, respectively. The diffusion coefficient is a function of the *r*^−1^, resulting in the diffusion-based separation that is efficient for small particles. In this study, an exosome separation chip was developed based on the dominant property of diffusion over sedimentation in nano-sized particles. We diffused bovine serum albumin (BSA) through the separation chip to confirm the separation efficiency and concluded that 6 h would be enough for separating the exosomes from the analyte. After that, exosomes from cell culture media and human serum were separated and analyzed.

## 2. Results

### 2.1. Diffusion-Based and Physical Stress-Free Extracellular Vesicle Separation

The sedimentation and diffusion of exosomes can be compared as follows. Considering that the density of serum and exosome were 1.024 g·mL^−1^ and 1.15~1.19 g·mL^−1^, respectively, and 1× PBS viscosity was 0.89 kg·ms^−1^, from the sedimentation equation, 150 nm-sized exosome gravitational sedimentation velocity was calculated as 4.62~6.09 × 10^−11^ m·s^−1^ or 4.00~5.26 × 10^−6^ m·day^−1^, and 50 nm-sized exosome gravitational sedimentation velocity was calculated as 5.14~6.77 × 10^−12^ m·s^−1^ or 4.44~5.85 × 10^−7^ m·day^−1^ [46]. This calculation conforms with typical ultracentrifugation time, which typically takes 4 h at 100,000× *g* [25,46]. On the other hand, from the Stokes–Einstein equation, coefficient D for 150 nm and 50 nm-sized exosome diffusion was calculated as 3.22 × 10^−14^ and 9.65 × 10^−14^ m^2^·s^−1^, respectively, in 1× PBS buffer at room temperature. The 1-dimensional diffusion length, i.e., $\sqrt{2Dt}$, for 150 nm-sized and 50 nm-sized particles/vesicles was 1.52 × 10^−5^ m for 1 h and 1.86 × 10^−5^ m for 30 min, respectively [45]. Considering that the PC membrane thickness was 1.5 × 10^−5^ m, 50~150 nm-sized exosomes can cross the membrane within 1 h, theoretically. For the above reason, it was concluded that the diffusive behavior of exosomes is dominant over gravitational sedimentation.

**Figure 1:**
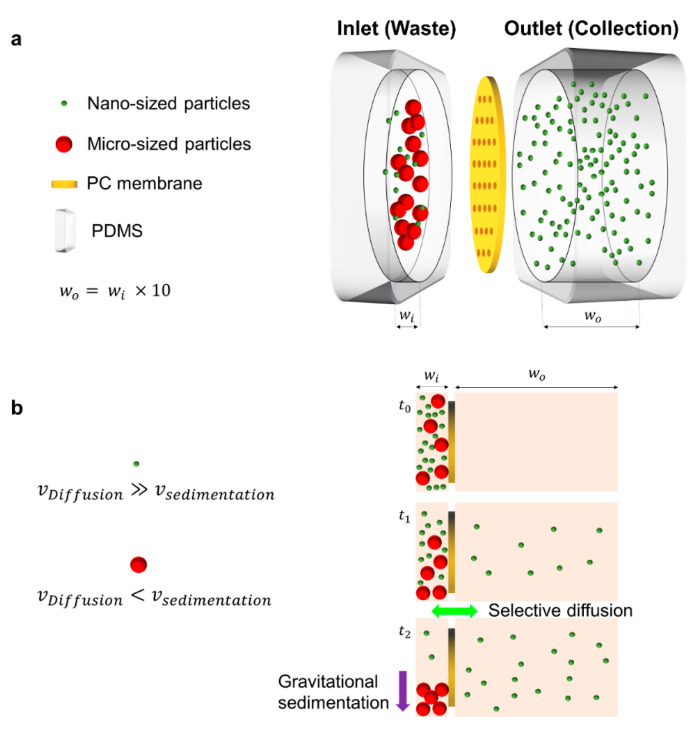
Schematic of the nanoporous PC membrane chip and its principle. (**a**) The chip is composed of a PC membrane with a diameter of 25 mm and two PDMS chambers that are inlet and outlet, respectively. PC membrane with a pore size of 200 nm is located vertically between the two chambers. The width of the outlet chamber (w_o_ = 5 mm) was designed to be 10 times larger than the width of the inlet chamber (w_i_ = 500 μm). (**b**) Nano-sized (shown in green color) and micro-sized (shown in red color) biomolecules show dominant movement of diffusion and sedimentation, respectively.

A nanoporous polycarbonate (PC) membrane chip was designed and fabricated to separate EVs based on their size dependency in diffusion coefficient and sedimentation velocity (Figure 1a and Figure S1). Nanoporous PC membrane, which acts as a filter for diffusion, was placed between two poly-dimethyl siloxane (PDMS) chambers. Particles or EVs larger than the pore size were unable to pass through the membrane, resulting in settling to the bottom of the inlet chamber. Smaller particles or EVs did pass through the membrane not only because they diffuse faster, but also because they would not sediment to the bottom of the inlet chamber (Figure 1b). The inlet chamber’s PDMS part has three pillars of 1 mm diameter, to prevent the chamber from collapsing due to the low aspect ratio (Figure S2). In the chip, the volume difference between the inlet and outlet chambers was designed to be 10-fold, to maximize the recovery of samples. This intentional volume difference ensured that 90% of the desired sample could be harvested by diffusion originated from concentration gradient.

### 2.2. Separation of Nano-Sized Materials

**Figure 2:**
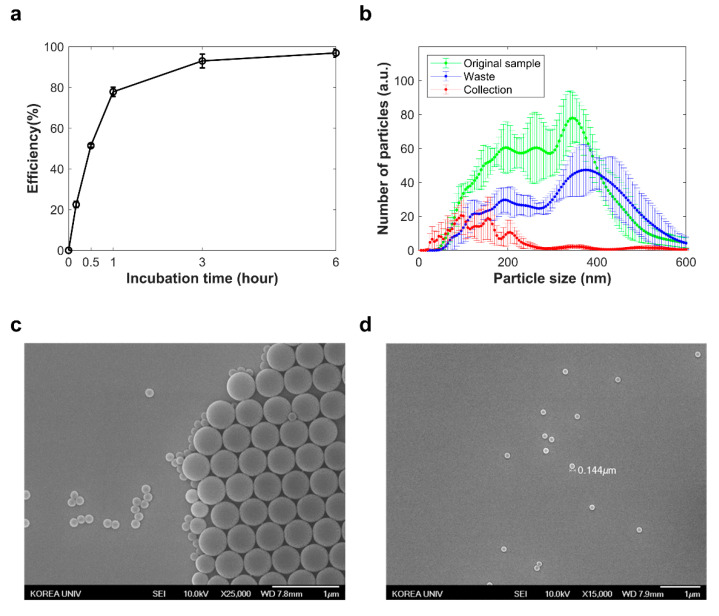
Separation results for nano-sized materials. (**a**) The separation efficiency of bovine serum albumin over time at the outlet chamber. (**b**) NTA result for polystyrene particle mixture before and after separation. (**c**) SEM image of the samples from inlet chamber and (**d**) outlet chamber.

To verify separation efficiency and determine optimal separation time, BSA with dimensions of 14 × 4 × 4 nm^3^ [47] was injected into the inlet chamber, and the concentration of BSA at the outlet chamber was measured over time by Bradford assay (Figure 2a). The outlet (collection) chamber’s Bradford assay signal was normalized relative to the Bradford assay signal at the theoretical concentration when BSA was sufficiently diffused and uniformly distributed in both chambers (inlet and outlet), and the separation efficiency was calculated. The separation efficiency reached 80% in 1 h. Besides the Bradford assay, two different-sized polystyrene particles of 132 nm and 500 nm were mixed, and the mixture was separated by the chip for 6 h. The NTA and SEM results showed that the major fraction of 132 nm and 500 nm particles were detected in the outlet and inlet chambers, respectively (Figure 2b–d). The nanoparticle separation efficiency was 38%.

### 2.3. Separation of EVs from Cell Culture Media

**Figure 3:**
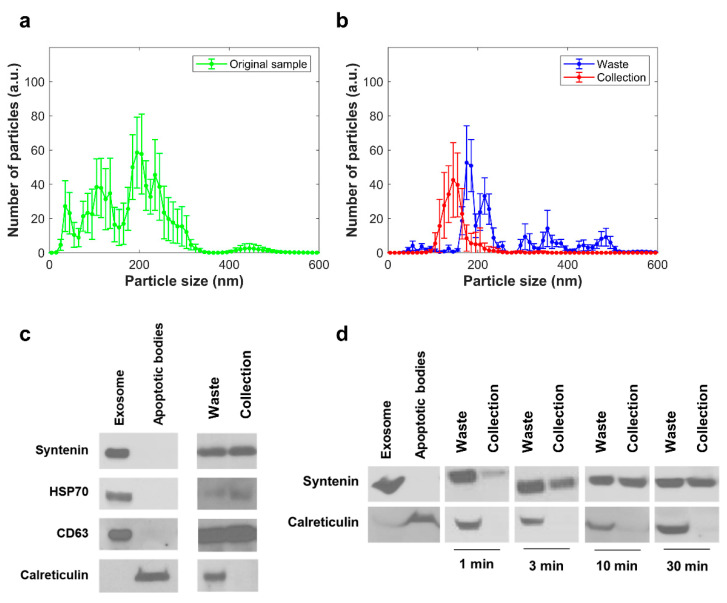
Results for separation of EVs from cell culture media. (**a**) NTA results for mixture of EVs and apoptotic bodies before separation. (**b**) NTA results for the two chambers after separation. (**c**) Immunoblotting results for the two chambers after 6 h of separation. Syntenin, HSP70, and CD63 are biomarkers of exosomes, and calreticulin is a biomarker of apoptotic bodies. (**d**) Immunoblotting results for time-dependent separation.

**Figure 4:**
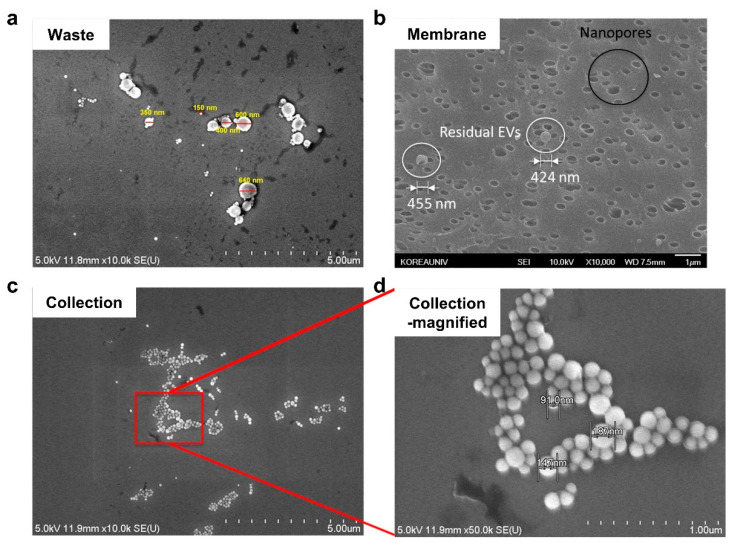
SEM image of separated EVs. (**a**) Sample from inlet shows both exosomes and apoptotic bodies. (**b**) Membrane after elution. Crater-like shapes in the upper black circle are nanopores; spherical features in white circles are apoptotic bodies, which could not pass through the membrane. (**c**) Sample from outlet chamber shows exosomes only. (**d**) Magnified view of the red box in (**c**).

Exosomes and apoptotic bodies were obtained from doxorubicin treated SW620 (Figure S3). NTA analysis showed effective size-dependent separation of the EVs with 200 nm pore size PC membrane (Figure 3a,b). At the outlet chamber, the size of the exosomes was found to be in the range of 50 to 150 nm, consistent with other reports [1]. The exosome recovery rate analyzed from NTA data was 42% for cell culture media samples. Exosome-specific markers, syntenin, HSP70, and CD63, were detected in both inlet and outlet chambers, while the apoptotic bodies-specific marker, calreticulin, was detected only in the inlet chamber after 6 h of separation (Figure 3c). Time-dependent separation was performed and verified by immunoblotting (Figure 3d). Samples at the outlet chamber were recovered and assayed after 1, 3, 10, 30 min of separation, while the separation time was measured from the injection of sample into the inlet chamber to sample recovery from each chamber. The SEM image shows exosomes were observed in the outlet chamber, while apoptotic bodies were only found in the inlet chamber of the nanoporous PC membrane chip (Figure 4). The SEM image suggests the physically intact morphology of the separated exosomes. Moreover, the biggest challenge when separating exosomes by ultrafiltration is clogging and trapping of vesicles on membrane [48]. Because pressure was not applied to the membrane during separation, the degree of clogging and trapping of exosomes in polycarbonate membrane was negligible (Figure 4b). In Figure 4b, only apoptotic bodies remain at the membrane, as they were larger than the pore size.

### 2.4. Separation of EVs from Blood

**Figure 5:**
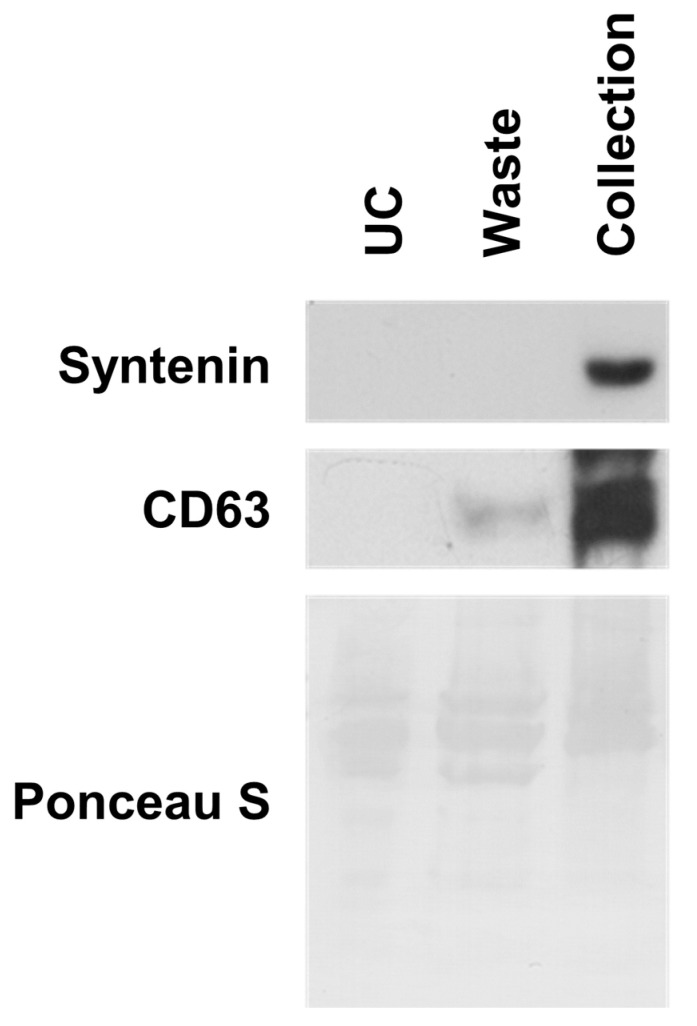
Separation of EVs from human serum using nanoporous PC membrane chip. Human serum EVs were separated by ultracentrifugation (UC) and nanoporous PC membrane chip. Original gel images are in Figures S7–S9.

When separating EVs from blood samples, proper adjustment of viscosity is important because the viscosity of the colloidal solution is an important factor in diffusion. The normal range for human serum viscosity is 3 to 4 centipoises, which is 3.3 to 4.5 times higher than that of 1× PBS [49]. So, we performed experiments to find optimal separation conditions for the blood samples. It was concluded that diluting the serum with PBS and including surfactant (0.005% (*w*/*w*) of Triton X-100) is required, and that the surfactant does not damage the exosomes (Tables S1 and S2, Figures S4 and S5) [50]. EVs from normal human off-clot serum were separated with the chip, and immunoblotting was conducted to compare the separation efficiency with ultracentrifugation. For this comparison, 150 µL of human serum and 150 µL of diluted human serum (1:1 with 1× PBS buffer of 0.01% (*w*/*w*) Triton X-100) were used for the ultracentrifuge- and diffusion-based separations, respectively, and the exosome isolation time for each method was 8 h. The diffusion-based separation with the small amount of sample showed a strong signal for both syntenin and CD63 antibodies (Figure 5). Especially, the negligible signal for syntenin in the waste indicated most of the exosomes translocated through the nanoporous membrane due to the 1:10 (waste:collection) volume ratio. However, the ultracentrifugation showed no separation signal because the loading amount of 150 µL was not enough to obtain an exosome pellet. For example, most studies on ultracentrifugation-based exosome separation used human serum samples of >1 mL and the recovery of exosome was low [39,51,52]. The ponceau S staining indicated that ultracentrifuge-based separation was conducted in the right manner (Figure 5). The high recovery rate for the diffusion-based separation with small volume of blood sample is advantageous for diagnosis because the blood sample needs to be shared among many analytical processes.

## 3. Discussion

A nanoporous PC membrane chip for size-dependent separation of EVs was developed on the basis of the fast diffusion of nanoscale particles. It can be developed in a variety of ways, such as with different pore sizes, chamber scales, etc. Using diffusion-based principles, various nano-sized particles/vesicles were successfully separated, including polystyrene particles and EVs, not only from cell-cultured media but also from serum. The diffusion coefficient increased as particle size decreased, and separation times were accordingly shortened. The chip is simple to use because it does not require any other external force or antibody, unlike other separation methods [53]. Together, these features resulted in chemically and physically stress-free separation of the exosome. In addition, with immunoaffinity-capturing methods, only EVs enriched with a specific marker could be isolated, leaving out many other EVs lacking the marker. We expect that the size-dependent separation method can balance this limitation.

Similar studies on EV separation using porous membranes had been performed previously. In previous microfluidic studies, an electric field of up to 200 V was perpendicularly applied to the microfluidic channel for the electrophoretic separation of negatively charged exosome [54,55]. Other filtration-based studies, utilizing two different sizes of porous PC membranes, demonstrated size-dependent EV separation using a syringe-filter-like setup [56,57]. In all of these previous filtration-based studies, voltage or vertical pressure (~5 bar) [58] was applied to the porous membrane for EV filtration through the PC membrane. Under these external forces, the filtered EVs are at risk of losing their physical intactness [59]. Compared to these studies, the chip developed in this study is more adequate to process ml scale separations, without any other electric or microfluidic experimental setup. However, no experimental approach for diffusion-based separation of EVs from biofluids has been reported. For EV-based therapeutics, a large-scale EV separation method is essential. Different EV separation methods, including ultracentrifugation, size exclusion chromatography, and microfluidics, are not easy to scale up. For example, microfluidics-based separation often requires hours of separation time for 1 mL (<16 µL·min^−1^) [60]. Compared to the one-dimensional structure of the microfluidic channel, the nanoporous PC membrane chip is two-dimensionally scalable simply by increasing the membrane area. As existing EVs separation methods, including ultracentrifugation, immunoaffinity separation, and micropillars, need complicated devices, instruments, processes, and skilled researchers, they show limitations of use in the clinical and pharmaceutical fields [35,61]. Diffusion-based separation does not require sample pre-processing; hence, the entire process of EV separation, injecting the sample into the inlet chamber and removing it from the outlet chamber, is simple, enabling unskilled operators to separate EVs using the chip. Despite the above advantages, diffusion-based separation might result in the dilution of EV. In addition, removing soluble proteins from the bio sample is an important issue in EV separation. To address this issue, we showed a sample concentration using a PC membrane chip with a pore diameter of 50 nm. The 50 nm diameter was chosen to be larger than soluble proteins and smaller than exosome. The chip design was modified by putting a syringe needle tip at the outlet chamber (Figure S6). A sample of 132 nm bead and bovine serum albumin (BSA) was injected through the access hole of the inlet chamber. As the inlet chamber filled up, the syringe was removed, concentrating only 132 nm bead in the inlet chamber while BSA was withdrawn to the outlet chamber. NTA results for the mixture before and after concentration show that it was possible to concentrate the remaining bead by 10×.

In summary, we report on a nanoporous PC membrane chip for EV separation using diffusion. This chip does not require any external force or antibody, guaranteeing that separated exosomes will be chemically and physically intact. The chip is easy to fabricate and handle and can be modified for other purposes. Because it does not require pre-processing of a sample, even unskilled personnel can use it for the separation of EVs from various samples.

## 4. Materials and Methods

### 4.1. Fabrication of the Nanoporous PC Membrane Chip

The chip fabrication was based on previous work [62]. Briefly, the inlet chamber was made using a micro-mold of photoresist SU-8 2075 (Microchem, Westborough, MA, USA). The photoresist was exposed to UV (365 nm, 21 mW cm^−2^) using an MU-60 exposure system (Cella Biotech, Korea) with a pattern mask and developed using AZ-1500 (AZ electronic materials, Branchburg, NJ, USA) (Figure S1a–f). PDMS fabrication was based on standard protocol [63]. PDMS (Dow corning, Midland, MI, USA) was mixed in a 10:1 ratio with curing agent Sylgard™ 184 (Dow corning, Midland, MI, USA). The outlet chamber was made of PDMS using an acrylic mold. Access holes of 4- and 8-mm diameter were devised for the inlet and outlet chambers, respectively (Figure S1g,h). Track-etch PC membrane of 25 mm diameter, Whatman^®^ Nuclepore 110606 (GE Healthcare, Chicago, IL, USA), with a pore size of 200 nm was placed between the 2 PDMS chambers. The two PDMS chambers were bonded with 50 W of oxygen plasma treatment (Femtoscience, Hwasung, Korea) for 1 min (Figure S1i,j).

### 4.2. Sample Injection and Separation

Before injecting samples, the inlet and outlet chambers were filled with 150 µL and 1.5 mL of PBS, respectively, for wetting the PC membrane. After 30 min, the inlet chamber PBS was replaced with 150 µL of a sample. The sample-injected chip was placed at room temperature during separation. Access holes for injecting the sample were sealed to prevent evaporation during the separation step.

### 4.3. Sample Preparation

Two types of samples were prepared. First, the polystyrene nanoparticle mixture solution was composed of particles of two different sizes: 132 nm (PS02N) (Bangs Laboratories, Fishers, IN, USA) and 500 nm (L3280) (Sigma-Aldrich, Saint Louis, MO, USA). Both sizes of particles have a coefficient of variation of 3% (*v*/*v*). Second, cell culture media-originated exosomes (colon cancer cell line, SW620 cells) were obtained (American Type Culture Collection, Manassas, VA, USA). Cells were cultured in RPMI 1640 medium, supplemented with 10% (*v*/*v*) fetal bovine serum and 1% (*v*/*v*) penicillin-streptomycin solution (Hyclone, Logan, UT, USA). Cells were maintained in a 5% CO_2_ incubator at 37 °C. To induce EVs from cells, they were treated with 2 μM doxorubicin hydrochloride (Sigma-Aldrich, Saint Louis, MO, USA). After 48 h of doxorubicin treatment, the culture medium was harvested and centrifuged at 400× *g* for 10 min and then at 10,000× *g* for 20 min, sequentially. Pellet was resuspended with PBS and then used as the sample of apoptotic bodies. The supernatant was ultra-centrifuged at 100,000× *g* for 180 min. The ultra-centrifuged pellet was then resuspended with PBS buffer and concentrated using Amicon centrifugal filter MWCO 10 kDa (Merk Millipore, Burlington, MA, USA). The mixture of EVs and apoptotic bodies was injected into the inlet chamber. Human serum (Zenbio, Durham, NC, USA) was purchased and used in separation after being centrifugated at 2000× *g* for 10 min. Serum was diluted 1:1 with a PBS buffer of 0.01% (*w*/*w*) Triton X-100 to prevent aggregation.

### 4.4. Sample Analysis

Samples were analyzed with Bradford assay, NTA, SEM, and immunoblotting. Bradford assay and nanoparticle tracking analysis were repeated 5 times. To verify separation efficiency over time, BSA solution (5 μg·mL^−1^) was used and the concentrations of the samples were measured by Bradford assay after the indicated time (10, 30, 60, 180, and 360 min). The efficiency was normalized by injected BSA amount. NTA machine Nanosight LM10 was used to measure the size distribution of the samples (Malvern Panalytical, Malvern, UK). For the measurement, 400 μL of the sample was loaded onto the sample stage at room temperature. For the SEM analysis of exosomes, samples were fixed as follows. The primary fixation was carried out with 2.5% (*w*/*w*) glutaraldehyde (Sigma-Aldrich, Saint Louis, MO, USA) in 0.1 M phosphate buffer (pH 7.4) for 4 h. Then, the samples were rinsed with 0.1 M phosphate buffer (pH 7.4) on a shaker for 10 min. After rinsing, the samples were fixed again with 1% (*w*/*w*) aqueous osmium tetroxide (Sigma-Aldrich, Saint Louis, MO, USA) in 0.1 M phosphate buffer (pH 7.4). For dehydration, samples were sequentially incubated with 25% (*v*/*v*) ethanol for 10 min, 50% (*v*/*v*) ethanol for 10 min, 75% (*v*/*v*) ethanol for 10 min, 95% (*v*/*v*) ethanol for 10 min, and 100% (*v*/*v*) ethanol for 10 min. After dehydration, the sample solutions were spin-coated on a wafer substrate at 2000 rpm for 60 s. The SEM imaging was carried out in two different instruments: for Figure 4a–c, S-4800 (Hitachi, Tokyo, Japan) was used; for Figure 2c,d, Figure 4b and Figure S4a–f, JSM-6701F (Jeol, Tokyo, Japan) was used. For the immunoblotting, samples were incubated with 15% (*v*/*v*) trichloroacetic acid (Sigma-Aldrich, Saint Louis, MO, USA) to precipitate exosomes, soluble proteins, and other vesicles. Then, the samples were precipitated with 10,000× *g* of centrifugation for 30 min. The pellet was then resuspended with pH 8.0, 0.1 M HEPES buffer (Sigma-Aldrich, Saint Louis, MO, USA). Samples were loaded onto SDS-PAGE and then transferred to PVDF membrane (Merck Millipore, Burlington, MA, USA). Anti-syntenin-1 (Santa Cruz Biotechnology, Dallas, TX, USA), CD63 (Invitrogen, Waltham, MA, USA), HSP 70 (Invitrogen, Waltham, MA, USA), and calreticulin (Invitrogen, Waltham, MA, USA) were used.

## Data Availability

Not applicable.

## Supplementary Materials

The following are available online at https://www.mdpi.com/article/10.3390/bios11090347/s1, Figure S1: Schematic representation of the fabrication process for nanoporous membrane chip, Figure S2: The nanoporous membrane chip, Figure S3: Schematic illustration of exosome preparation from cell culture media, Figure S4: SEM images of dried outlet solution under each separation condition, Figure S5: NTA results for Table S2. Figure S6: Methodology for removing soluble proteins from the sample, Figure S7: Original gel images for Figure 3c, Figure S8: Original gel images for Figure 3d, Figure S9: Original gel images for Figure 5, Table S1: Expected diffusivity and final concentration change according to the dilution ratio, Table S2: Tested conditions.
