# Supplementary material for: Diffusion-Based Separation of Extracellular Vesicles by Nanoporous Membrane Chip
Source: Biosensors (Basel). 2021 Sep 19;11(9):347. doi: 10.3390/bios11090347 (PMC8472239; doi:10.3390/bios11090347)
Supplement: Supplementary file 1 [file biosensors-11-00347-s001.zip › biosensors-1355444-supplementary.pdf]

biosensors

MDPI

Article

Diffusion-Based Separation of Extracellular Vesicles by Nanoporous Membrane Chip

Gijung Kim [1,†], Min Chul Park [2,†,‡], Seonae Jang [3], Daeyoung Han [2], Hojun Kim [1], Wonjune Kim [1], Honggu Chun [1,4,*] and Sunghoon Kim [2,5,6*]

[1] Department of Biomedical Engineering, Korea University, Seoul 02841, Korea; novelty@korea.ac.kr (G.K.); hjkim1017@korea.ac.kr (H.K.); aether26@korea.ac.kr (W.K.)
[2] Medicinal Bioconvergence Research Center, Gyeonggido 16229, Korea; minchul.park@neomics.com (M.C.P.); ladin20@snu.ac.kr (D.H.)
[3] Department of Biomicrosystem Technology, Korea University, Seoul 02841, Korea; suneai87@hanmail.net
[4] Interdisciplinary Program in Precision Public Health, Korea University, Seoul 02841, Korea
[5] Institute for Artificial Intelligence and Biomedical Research, Seoul 03722, Korea
[6] College of Pharmacy & College of Medicine, Gangnam Severance Hospital, Yonsei University, Seoul 03722, Korea
* Correspondence: chunhonggu@korea.ac.kr (H.C.) and sunghoonkim@yonsei.ac.kr (S.K.)
† These authors contributed equally.
‡ Present address: Curebio Therapeutics Co., Ltd., Suwon 16229, Korea.

Supporting information:

Section 1. The fabrication process of nanoporous membrane chip

Section 2. Exosome sample preparation and determination of serum solution condition

Section 3. Removing soluble proteins from the sample

Section 4. Original gel images of immunoblot

Section 1. The fabrication process of nanoporous membrane chip

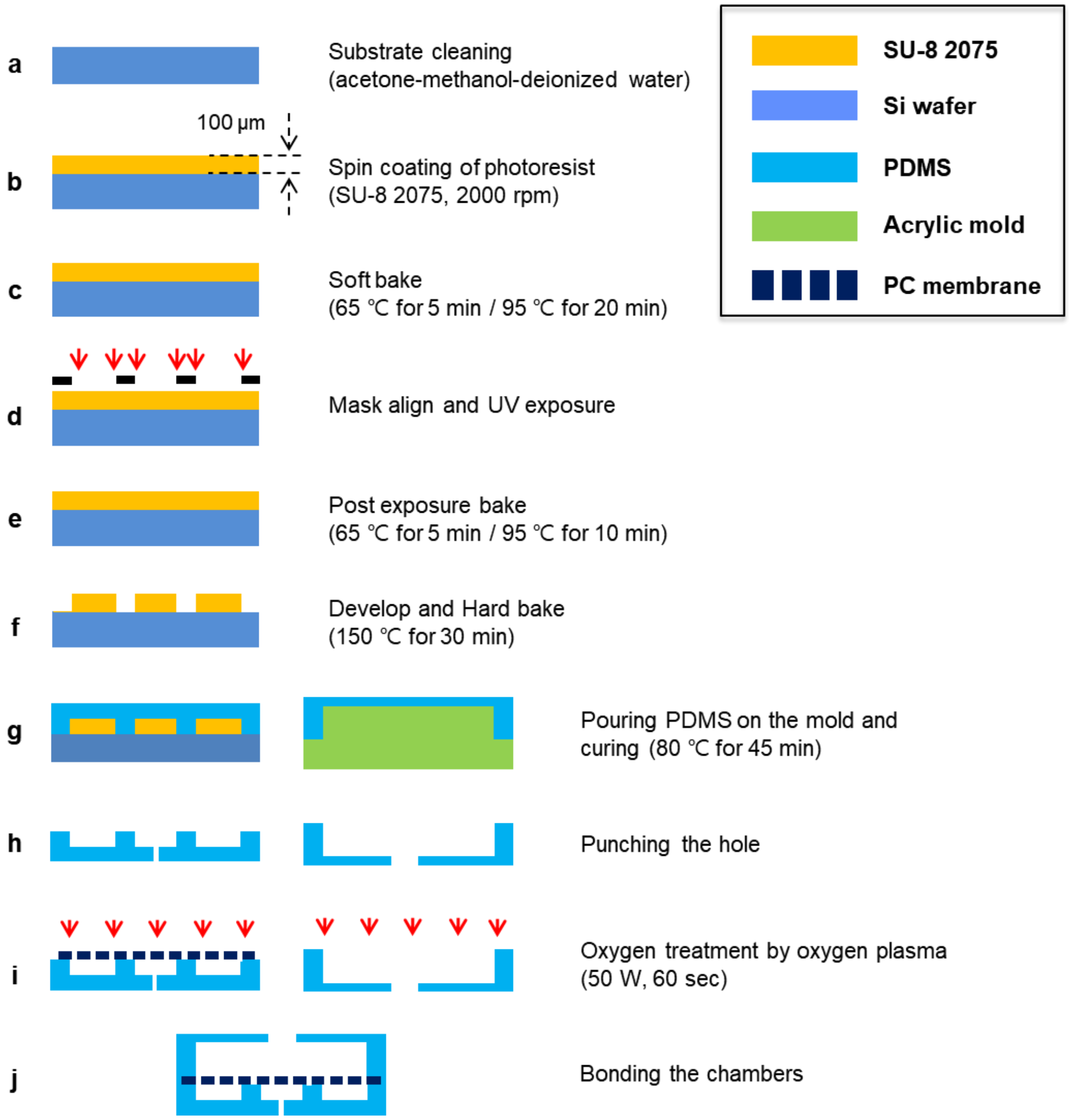

Figure S1. Schematic representation for the fabrication process of nanoporous membrane chip. (a) 4′′ silicon wafer prepared. (b) Photoresist spin-coated. (c) PR soft baked. (d) UV light applied and patterned with mask aligner. (e) PR post-exposure baked. (f) PR development and hard-baked. (g) PDMS pre-polymer poured and cured on the master molds. (the left and the right figure represents mixture inlet and sample outlet, respectively) (h) PDMS punched. (i) Oxygen plasma treatment. (j) Bonding two PDMS and the PC membrane.

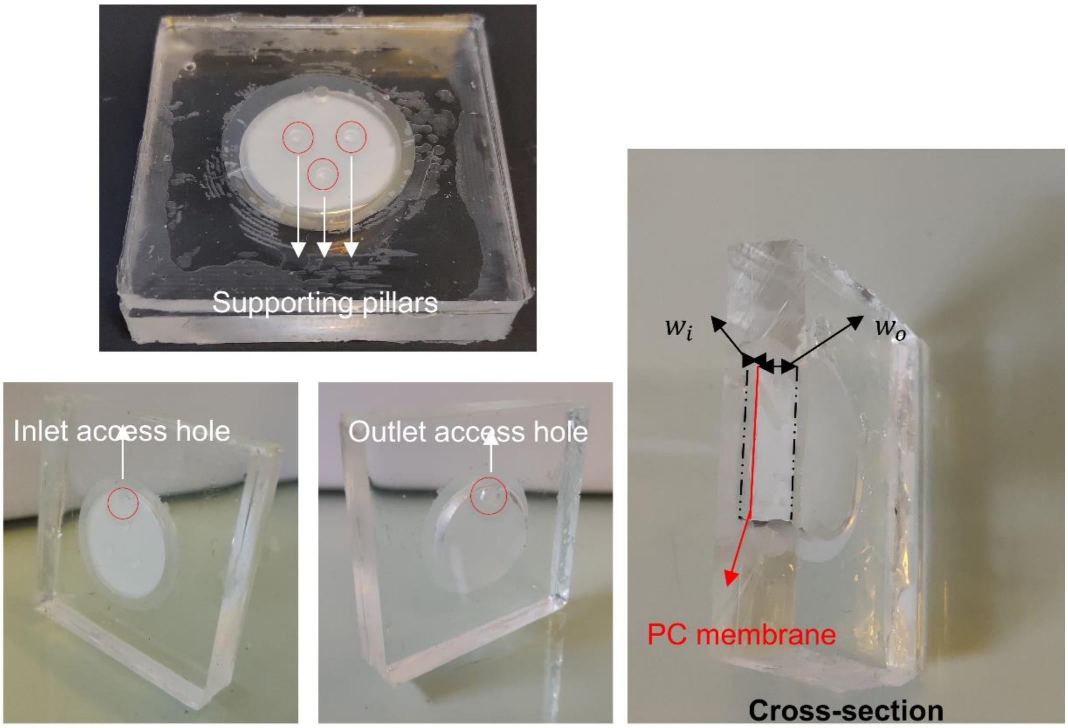

Figure S2. The nanoporous membrane chip. The chip has a different ratio of inlet chamber and outlet chamber (10x volume), resulting in efficient separation.

Section 2. Exosome sample preparation and determination of serum solution condition

2.1. Exosome preparation from cell media

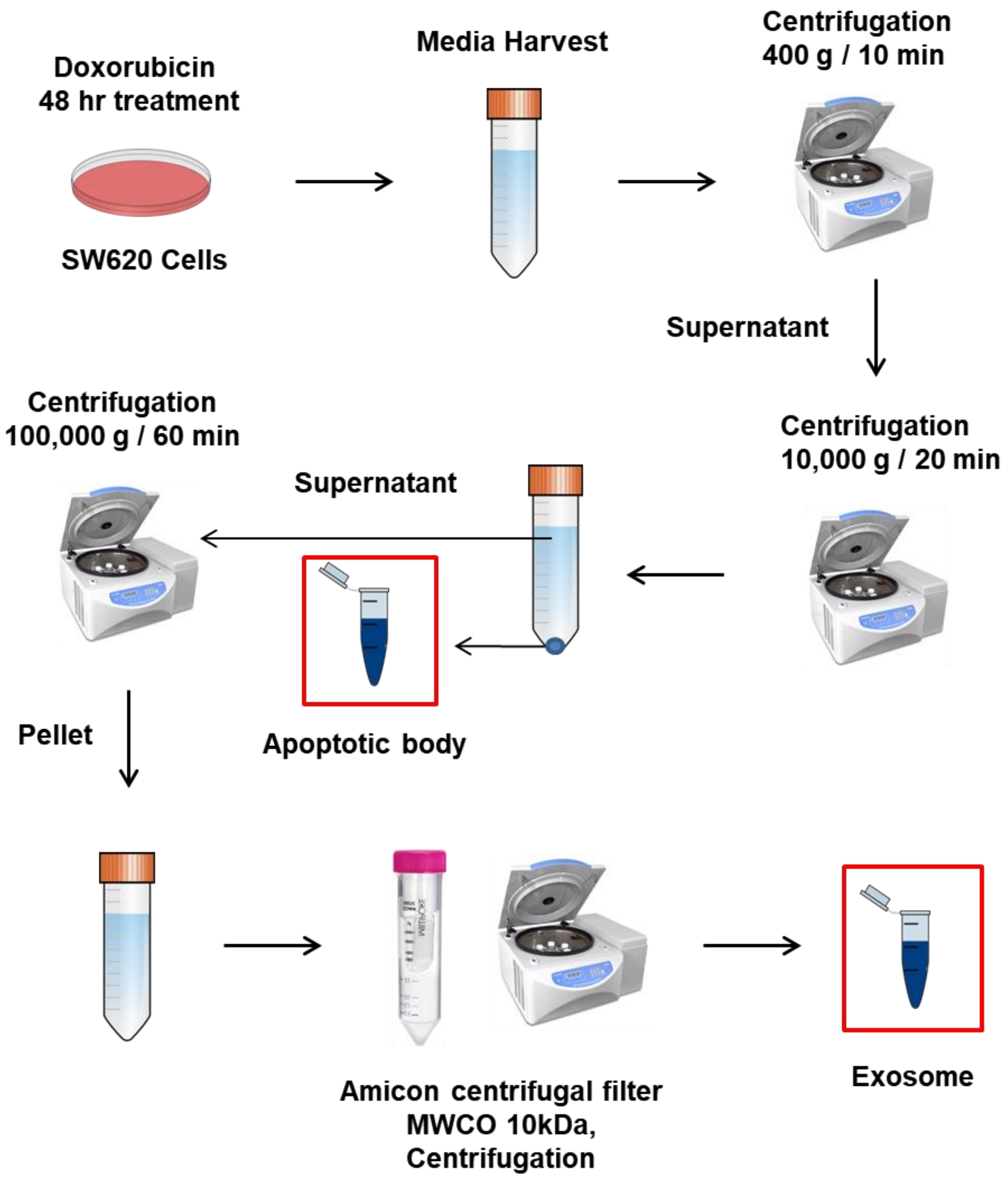

Figure S3. Schematic illustration of exosome preparation from cell culture media. Colon cancer cell line, SW620, was used to obtain the exosome and apoptotic body. Exosome and apoptotic body, induced by doxorubicin (2 μM), were isolated by serial centrifugation.

2.2. Determination of serum solution condition

The higher viscosity of sample solution results in slower diffusion of particles, which will lower separation efficiency. Most exosome-containing samples such as cell culture media or serum are highly viscous due to soluble proteins or lipids, therefore, it is essential to control viscosity. There are two methods to decrease viscosity. One is to dilute the sample with less viscous solutions and the other is to add surfactant. In this section, the dilution factor and the effects of optimal surfactant for exosomes separation are discussed. The calculated viscosity of two different solutions follows the equation:

$$\ln \eta_m = a \ln \eta_i + b \ln \eta_d$$

where η_m is the dynamic viscosity of solution after mixed, η_i is the dynamic viscosity of solution for the original solution, η_d is the dynamic viscosity of diluting solution, and a, b is the volume ratio of each solution to the total mixed volume (Grunberg, L et al Nature 164.4175 (1949): 799-800). Diluting serum sample has a trade-off. It helps the diffusion of nanoparticles by lowering viscosity, but the final concentration of the separated sample will be lower. Keeping this in mind, we can make a table regarding viscosity change, diffusivity change, and exosome concentration change according to the ratio of 1X PBS to serum (Table S1).

1X PBS to Serum ratio	0.25	0.43	0.67	1	1.5	2
Ratio of change in viscosity	1.56	1.46	1.36	1.27	1.18	1.13
Ratio of change in diffusivity	1.15	1.24	1.33	1.42	1.53	1.60
Ratio of change in exosome concentration	0.8	0.7	0.6	0.5	0.4	0.3

Table S1. Expected diffusivity and final concentration change according to the dilution ratio. Diluting the sample will help the separation, while we should consider its double-sided diluting effect on the concentration of exosomes.

Several types of surfactants are available to lower the viscosity, while only a few of them can be used considering our purpose of the EVs separation. Ionic surfactant is not appropriate for exosome separation, because commonly used ionic surfactant, such as sodium dodecyl sulfate (SDS), disrupts the membrane and denatures proteins easily. On the contrary, nonionic surfactants are considered mild surfactants. We tested two famous nonionic surfactants, Tween 20 or Triton X-100, since they are frequently used in most biology laboratories. The surfactant's concentration, 5 % of Tween 20 and 0.01 % Triton X-100, is the minimum concentration that does not disrupt EVs, from a previously reported study (Osteikoetxea, Xabier, et al. Organic & biomolecular chemistry 13.38 (2015): 9775-9782). The following table shows tested conditions for finding better diffusion conditions of human blood serum using 30 nm carboxylate polystyrene beads (Table S2). Both dilution of serum with 1X PBS and using a certain amount of surfactant was tested. Unlike water-based solutions such as 1X PBS, blood serum has low accuracy when measured by NTA because serum contains various materials. Therefore, we validate the efficacy at each condition based on the SEM image. The 30 µL of the polystyrene bead solution with 1.68 × 10^{15} particles/mL was added for each condition. After finishing the diffusion of the beads across the membrane, 10 µL of solution from the outlet chamber will contain 2.35 × 10^{11} beads. The total surface area of this number of 30 nm beads will be 1.66 × 10^{8} µm^2.

#	Volume (µL)

	**Condition description**	Human serum	1X PBS	Tween 20	Diluted Triton X-100	Bead solution
**1**	Normal serum	150	-	-	-	30
2	Only dilution	90	60	-	-	30
3	Only Triton X-100	148	-	-	2	30
4	Only Tween 20	143	-	7	-	30
5	Dilution and Triton X-100	90	58	-	2	30
6	Dilution and Tween 20	90	53	7	-	30

Table S2. Tested conditions. All samples were made to be 180 μL in total, and 150 μL of each sample was separated.

After 6 hours of separation, 10 μL from the sample outlet chamber was recovered and dried under the clean bench for 30 minutes. The SEM images of dried spots were then taken at the edge of the spots considering the coffee ring effect. Throughout the SEM imaging, we concluded that both diluting and using surfactants is the best condition to separate nanoparticles (Figure S4 e, f). Some beads were also found in all other conditions (Figure S4 a-d). However, the total number of beads was much smaller than both diluted and surfactant-used conditions.

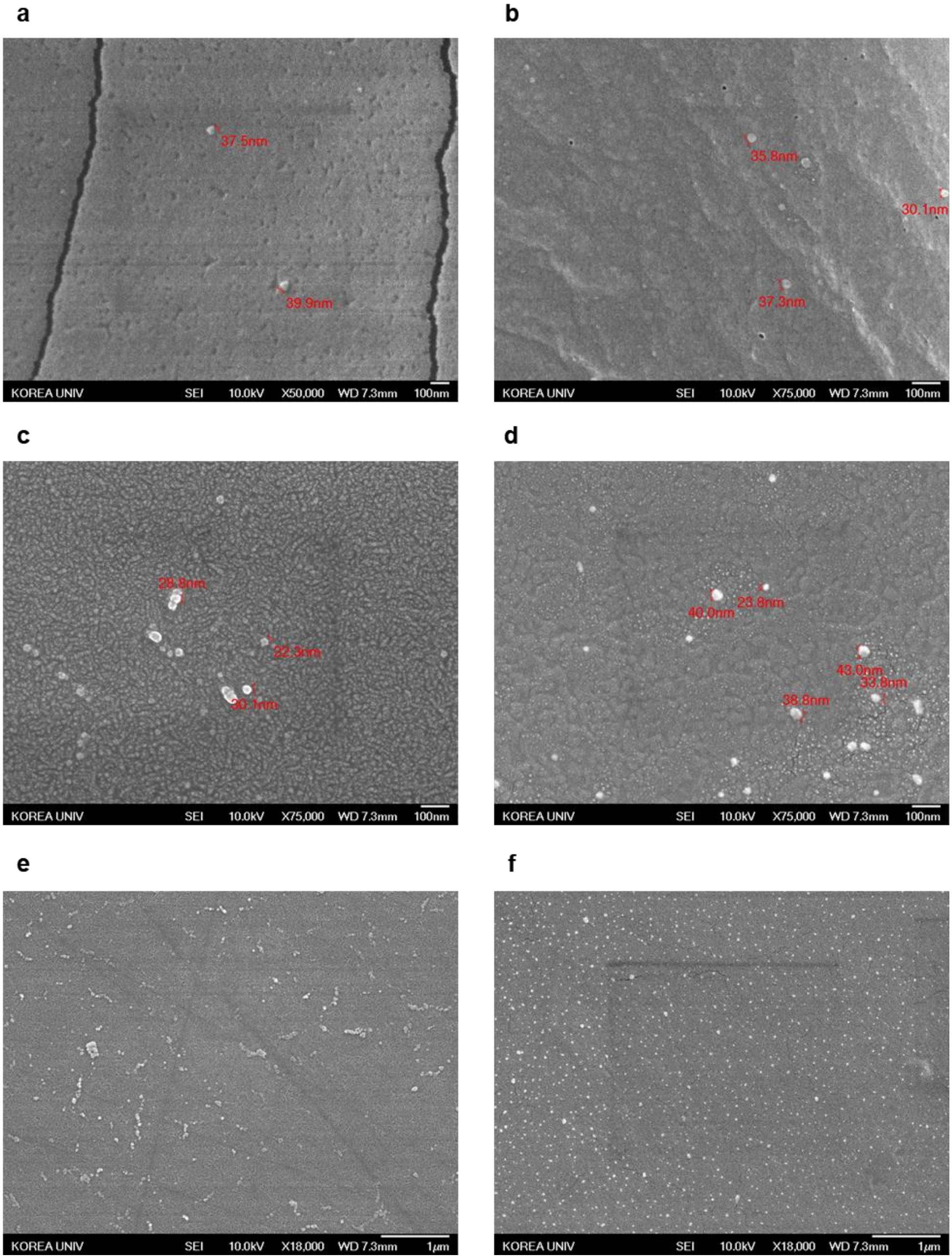

Figure S4. SEM images of dried outlet solution at each separation condition. After separation, 10 μL of each sample from the outlet chamber was dried and the SEM images were taken. (a) Separated result of normal serum condition. (b) Separated result of only dilution condition. (c) Separated result of only Triton X-100 condition. (d) Separated result of only Tween 20 condition. (e) Separated result of both diluted and Triton X-100 condition. (f) Separated result of both diluted and Tween 20 condition.

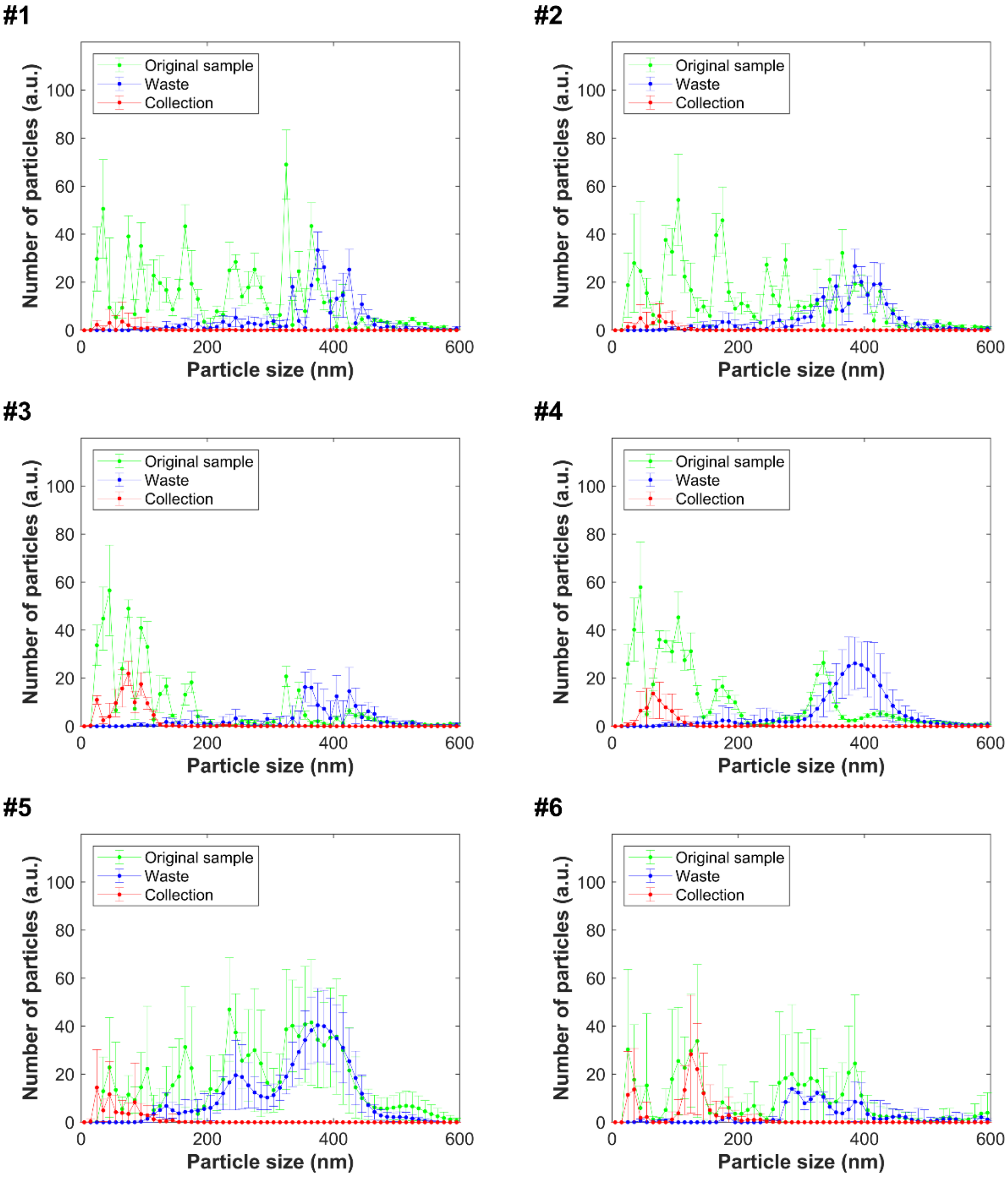

Figure S5. NTA results of Table S2. Recovery rates of #1 and #2 is under 5%, recovery rates of #3 and #4 is around 18%, and recovery rates of #5 and #6 is 55% and 49%, respectively.

Section 3. Removing soluble proteins from the sample

We present a simple methodology utilizing a similar setup to isolate EVs from soluble proteins. Here we showed 132 nm bead in BSA solution got concentrated using 50 nm PC membrane. Note that the access hole of the outlet chamber is closed in this experiment.

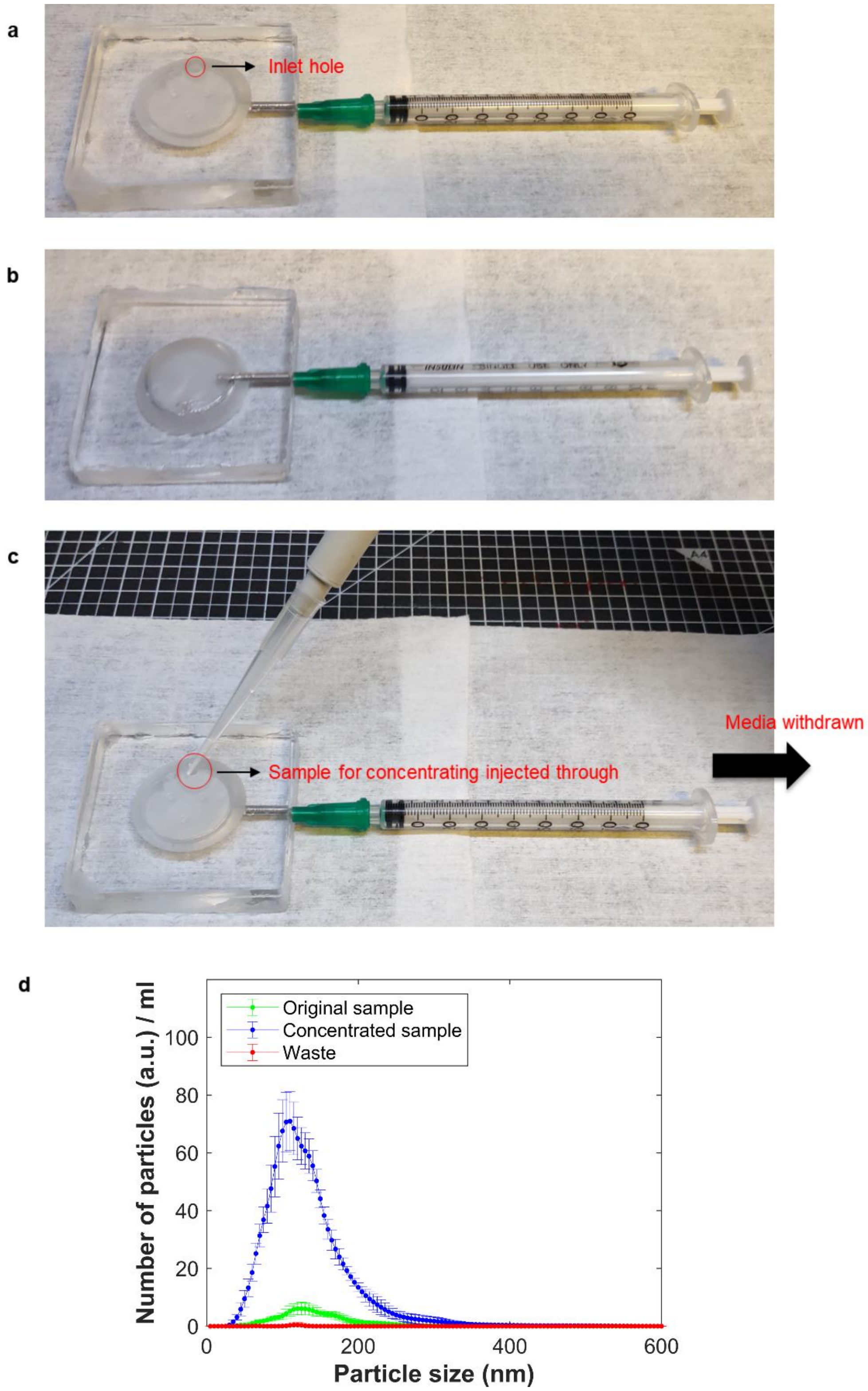

Figure S6. Methodology for removing soluble proteins from the sample. (a) The same design of the nanoporous membrane chip, which has different sizes of the pore, was modified by putting a

syringe needle tip at the outlet chamber. This image shows inlet chamber is upward. (b) This image shows inlet chamber is downward. Note that the access hole of the outlet chamber is closed in this experiment. (c) Sample of 132 nm bead and BSA was injected through access hole of the inlet chamber. As the inlet chamber gets filled, the syringe was pulled accordingly, concentrating only 132 nm bead in the inlet chamber while BSA gets withdrawn to the outlet chamber and syringe. (d) NTA results of bead and BSA mixture before concentration. (e) NTA results of remaining solution in the inlet chamber after 1.5 mL of sample was concentrated to ten times.

Section 4. Original gel images of immunoblot

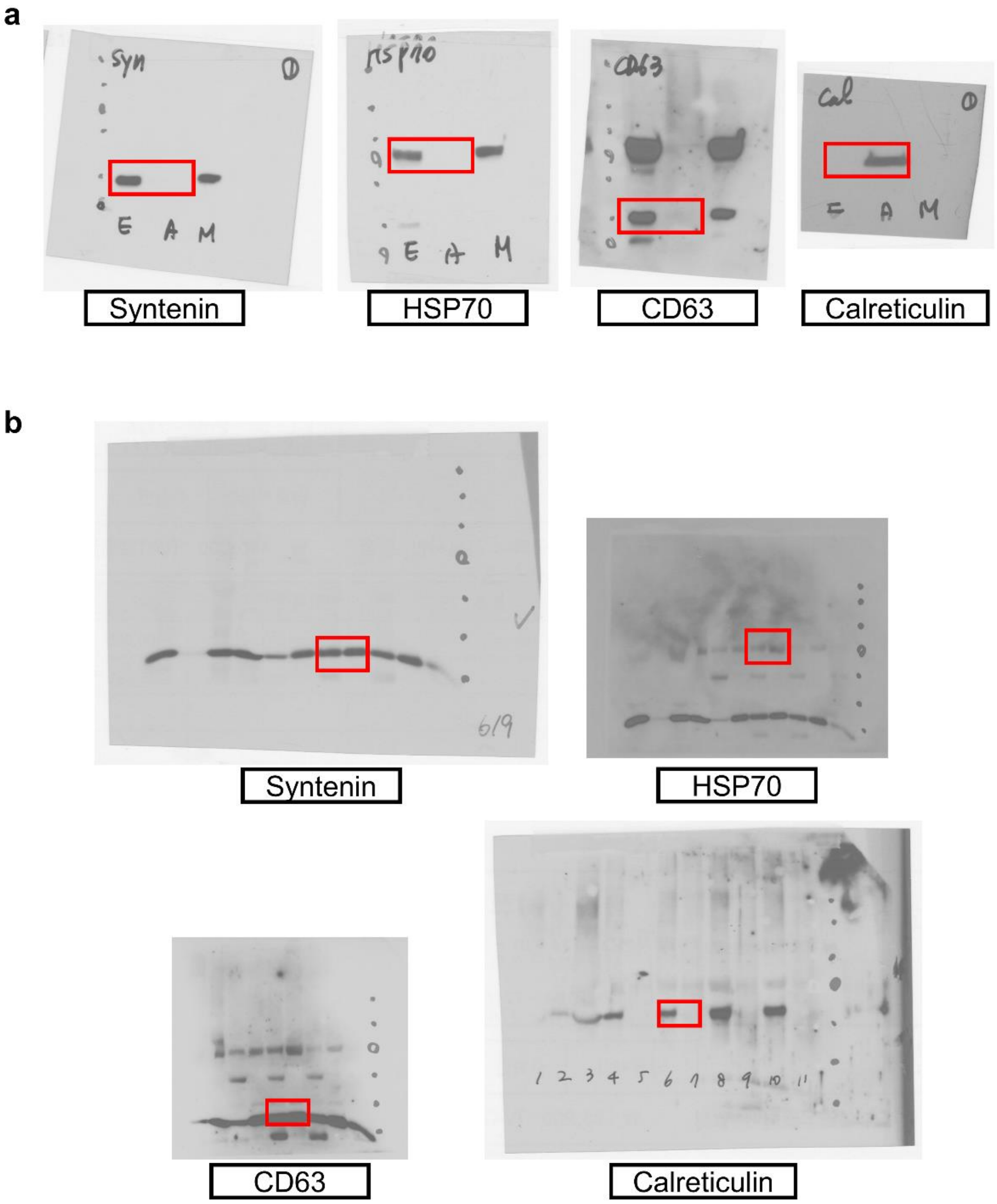

Figure S7. Original gel images for figure 3c. (a) figure 3c-left column-each gel result in the red box represents exosome and apoptotic bodies, respectively. (b) figure 3c-right column-each gel result in the red box represents inlet/outlet, respectively.

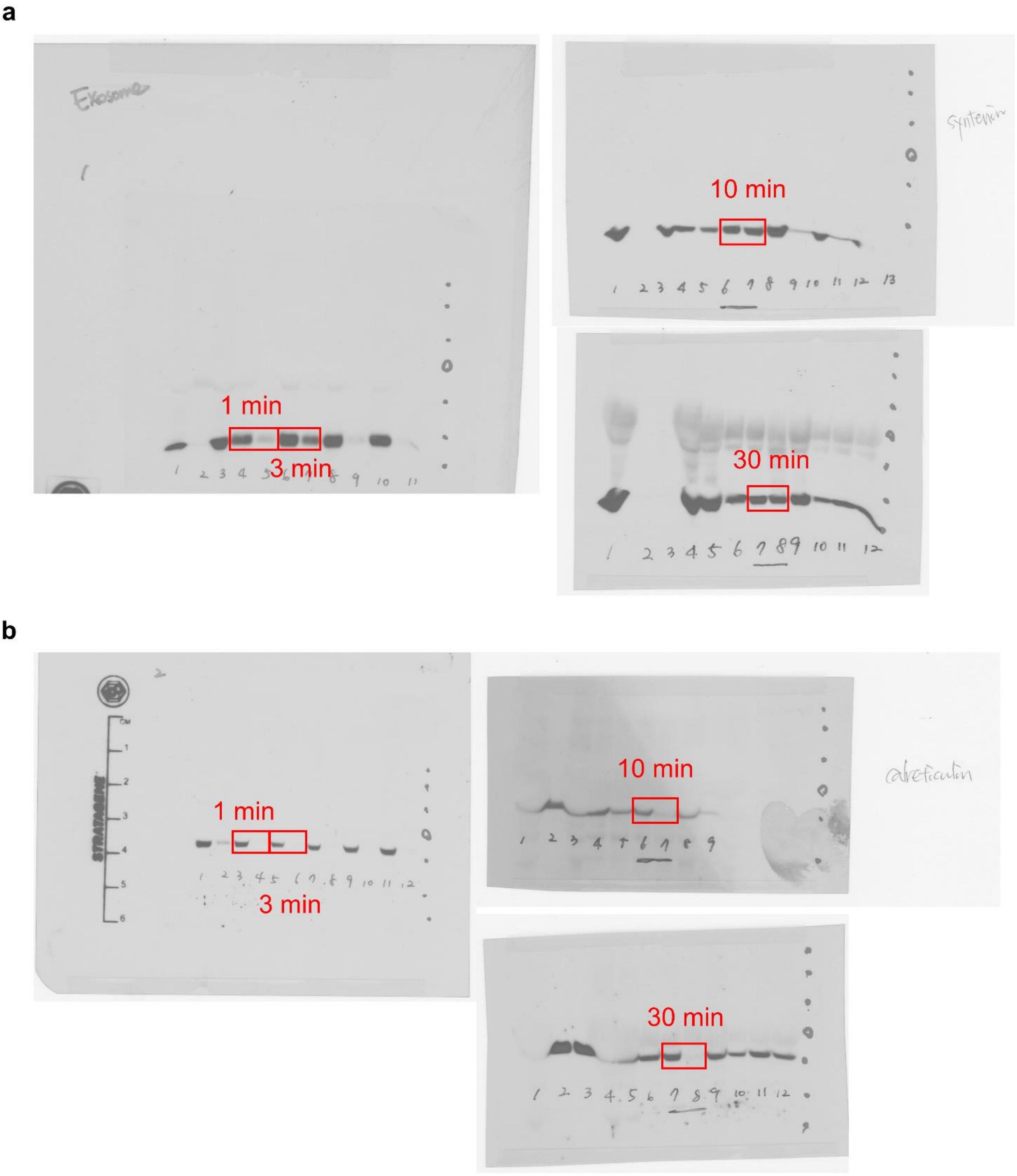

Figure S8. Original gel images for figure 3d. (a) Original gel result targeting Syntenin. (b) Original gel result targeting Calreticulin.

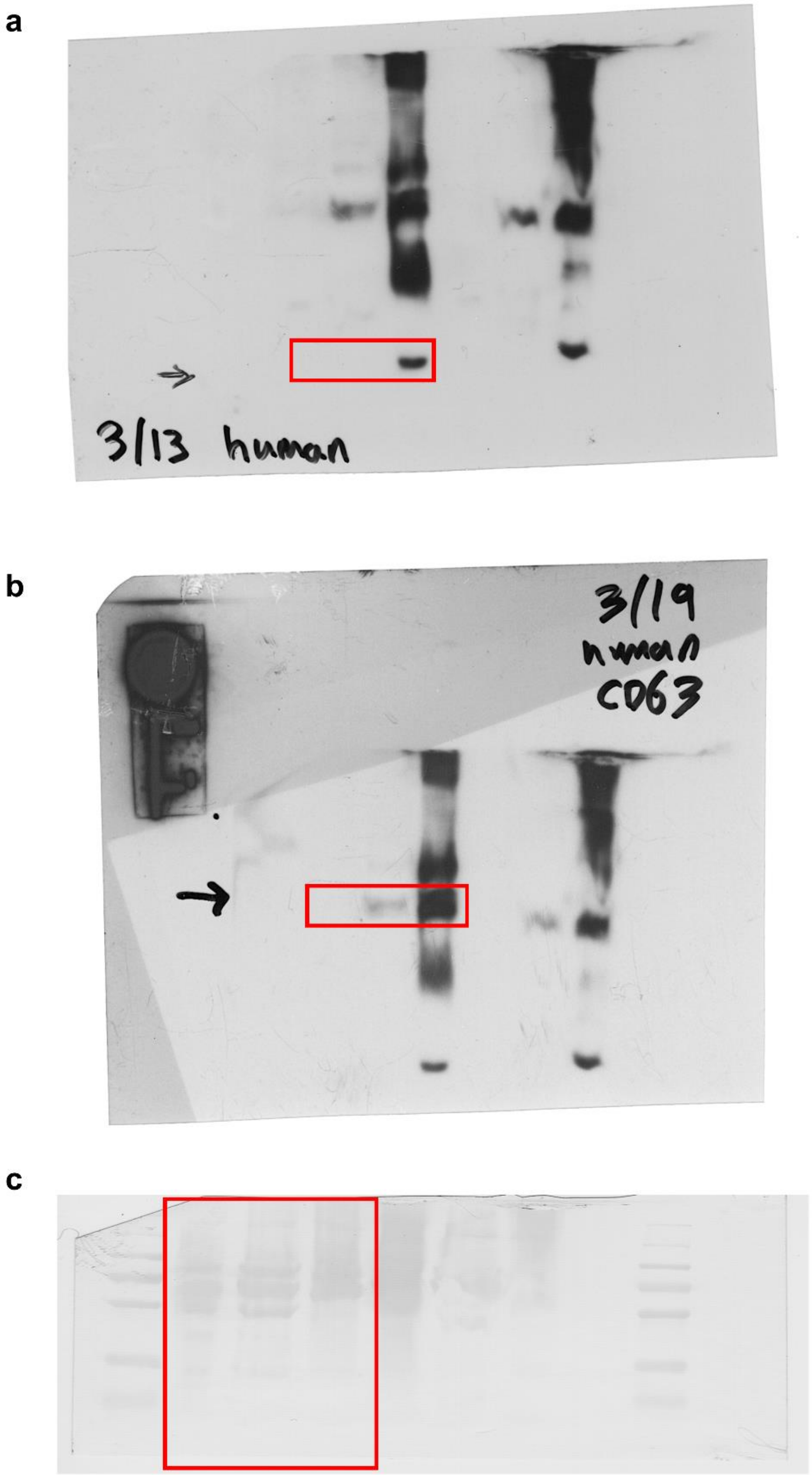

Figure S9. Original gel images for figure 5. Each gel result in the red box represents UC, inlet, and outlet, sequentially. (a) Original gel result targeting Syntenin. (b) Original gel result targeting CD63. (c) Original gel result targeting Ponceau S.
